# The Secreted Metalloprotease ADAMTS20 Is Required for Melanoblast Survival

**DOI:** 10.1371/journal.pgen.1000003

**Published:** 2008-02-29

**Authors:** Debra L. Silver, Ling Hou, Robert Somerville, Mary E. Young, Suneel S. Apte, William J. Pavan

**Affiliations:** 1 Genetic Disease Research Branch, National Human Genome Research Institute, Bethesda, Maryland, United States of America; 2 Department of Biomedical Engineering, Cleveland Clinic Foundation-ND20, Cleveland, Ohio, United States of America; Stanford University School of Medicine, United States of America

## Abstract

ADAMTS20 (A disintegrin-like and metalloprotease domain with thrombospondin type-1 motifs) is a member of a family of secreted metalloproteases that can process a variety of extracellular matrix (ECM) components and secreted molecules. *Adamts20* mutations in *belted* (*bt*) mice cause white spotting of the dorsal and ventral torso, indicative of defective neural crest (NC)-derived melanoblast development. The expression pattern of *Adamts20* in dermal mesenchymal cells adjacent to migrating melanoblasts led us to initially propose that *Adamts20* regulated melanoblast migration. However, using a *Dct*-LacZ transgene to track melanoblast development, we determined that melanoblasts were distributed normally in whole mount E12.5 *bt/bt* embryos, but were specifically reduced in the trunk of E13.5 *bt/bt* embryos due to a seven-fold higher rate of apoptosis. The melanoblast defect was exacerbated in newborn skin and embryos from *bt/bt* animals that were also haploinsufficient for *Adamts9*, a close homolog of *Adamts20*, indicating that these metalloproteases functionally overlap in melanoblast development. We identified two potential mechanisms by which *Adamts20* may regulate melanoblast survival. First, skin explant cultures demonstrated that *Adamts20* was required for melanoblasts to respond to soluble Kit ligand (sKitl). In support of this requirement, *bt/bt;Kit^tm1Alf^*/+ and *bt/bt;Kitl^Sl^*/+ mice exhibited synergistically increased spotting. Second, ADAMTS20 cleaved the aggregating proteoglycan versican *in vitro* and was necessary for versican processing *in vivo*, raising the possibility that versican can participate in melanoblast development. These findings reveal previously unrecognized roles for *Adamts* proteases in cell survival and in mediating Kit signaling during melanoblast colonization of the skin. Our results have implications not only for understanding mechanisms of NC-derived melanoblast development but also provide insights on novel biological functions of secreted metalloproteases.

## Author Summary

Mice with black and white coat coloration patterns have long been favorites of mouse fanciers and geneticists alike. Analysis of mouse coat color mutants has yielded important insights into normal developmental pathways as well as human disease processes. In this study we have investigated how mutations in a secreted metalloprotease, *Adamts20,* result in mice with white belts in their lumbar region, even though *Adamts20* is not expressed in the pigment producing cells. Our findings suggest that the belting pattern is due to a combination of increased pigment cell death, decreased pigment cell number in the trunk, and functional overlap of closely related metalloproteases. *Adamts20* mutants have disrupted function of Kit, a protein that regulates pigment cell development, as well as alterations in the extracellular matrix that surrounds the pigment cells. These findings have implications both for our understanding of general mechanisms of pigment cell development as well as for new biological functions of secreted metalloproteases.

## Introduction

A disintegrin-like and metalloprotease with thrombospondin type-1 motifs (ADAMTS) metalloproteases constitute a large family of 19 zinc-dependent proteolytic enzymes that are distantly related to both the A disintegrin and metalloproteinase (ADAM) family, and to the matrix metalloproteinases (MMPs) [1],[2]. In contrast to the ADAM proteases that are membrane anchored, ADAMTS proteases are secreted; however, some may be considered as operational cell-surface proteases as they bind to the cell surface. Some ADAMTS proteases, such as ADAMTS10 (GeneID: 224697), ADAMTS13 (GeneID: 279028), and the procollagen amino-propeptidases (e.g. ADAMTS2), are highly specialized; others process a variety of substrates within the extracellular matrix (ECM), including chondroitin sulfate proteoglycans (CSPGs), such as aggrecan (GeneID: 11595) and versican (GeneID: 13003). Mouse models harboring mutations in ADAMTS family members have demonstrated the importance of these proteases during development. In some cases mutant phenotypes can be attributed to a failure to cleave specific substrates [3]–[7]. Mutation or dysregulation of ADAMTS proteases is associated with inherited and acquired pathologies including Ehlers-Danlos syndrome VIIC (OMIM#225410), thrombocytopenic purpura (OMIM#274150), Weill-Marchesani syndrome (OMIM#277600) and arthritis [5], [8]–[10].

Among the animal mutants in *Adamts* proteases is a classical white-spotted mouse named *belted* (*bt*), so named because it contains white spots in the lumbar region creating the appearance of a belt [11]–[13]. Sequencing of three of the twelve known alleles of *belted*—*bt^Bei1^*, *bt^Mri1^*, and *bt* (Mouse Genome Informatics, MGI: 2660628)—revealed nonsense or missense mutations in *Adamts20*, thus implicating metalloproteases in skin pigmentation [12]. Analyses of white spotting mutants suggest that such phenotypes are often due to defective development of neural crest (NC)-derived melanoblasts that produce pigment of the integument (skin, hair, feathers, and scales), inner ear, and eye [14],[15].

Melanoblasts develop from a subset of NC that emigrate from the neural tube and overlying ectoderm and migrate dorso-laterally relative to the neural tube through prospective dermal mesenchyme (embryonic day (E) 8.5-E9.5) [15],[16]. Subsequently, the melanoblasts differentiate and expand (E9.5-E13.5), migrate into the epidermis and hair follicle (E13.5-E15.5), and eventually produce melanin (E15.5-P0). Several molecules, including the receptor tyrosine kinase Kit (GeneID: 16590) and its ligand, Kit ligand (Kitl, GeneID: 17311), regulate melanoblast development. *Kit* and *Kitl* act throughout melanoblast development, with independent requirements for melanoblast survival, proliferation and migration [17]–[23]. *Kit* is expressed on melanoblasts, and *Kitl* is expressed in the dermis and in dermal mesenchymal condensations and papillae [21], [24]–[26].

Similar to *Kitl*, *Adamts20* is expressed in dermal mesenchymal cells adjacent to and in advance of migrating melanoblasts throughout their development. This expression pattern led us to initially propose that the *bt* phenotype was caused by defective melanoblast migration [12]. This hypothesis was also based upon the observation that the *Adamts20* ortholog *Gon-1* (GeneID: 177850) is essential for gonadal morphogenesis and distal tip cell migration in *C.elegans* [27]–[29]. The current study tests our hypothesis by performing extensive characterization of melanoblast development in *bt/bt* embryos. Our findings suggest that *Adamts20* mutant mice exhibit white spotting in a belted pattern due to a combination of regional variation of melanoblast number, increased apoptosis, and functional overlap with *Adamts9* (GeneID: 101401). Furthermore, our analyses of *bt/bt* embryos indicate that defective Kit signaling and versican processing may explain the failure of melanoblasts to develop properly.

## Results

### Genetic and Molecular Characterization of a New *Belted* Allele

**Figure 1:**
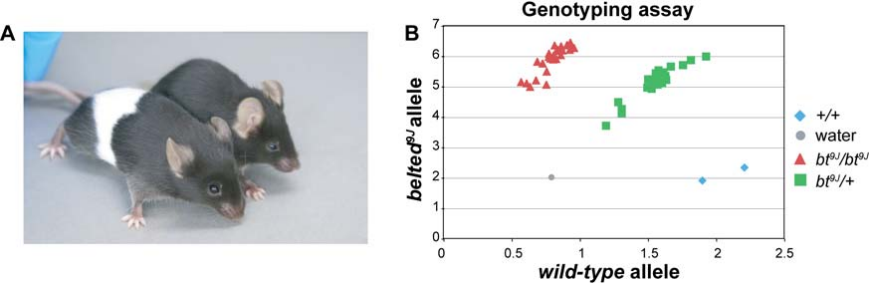
The *bt^9J^/bt^9J^* mouse contains a mutation in *Adamts20*. (A) An image of a *bt^9J^/bt^9J^* mouse (left) and a C57BL/6 mouse (right). (B) A representative result from a genotyping assay for the following genotypes: *bt^9J^*/*bt^9J^* (red triangle), *bt^9J^*/+ (green square), *wild-type* (C57BL/6) (blue diamond), and water control (grey circle).

*Adamts20* mutant mice exhibit white spotting in the lumbar region on both dorsal and ventral surfaces, frequently resulting in the appearance of a white belt in recessive animals (Figure 1A) [11]–[13]. For these studies an allele of *bt* on an inbred C57BL/6 background was used, as different genetic backgrounds can affect the expressivity of the *bt* phenotype [30],[31]. Since an *Adamts20* mutation had not been specifically demonstrated in this particular *bt* allele, a complementation cross was performed between these *bt* mice and *bt^Bei1^/+* mice. Seven out of 10 animals born exhibited a *bt* phenotype, consistent with the expected Mendelian ratios (50% *bt*) and indicative of a recessive allele of *bt*. This allele has now been designated *bt^9J^* and is listed at MGI.

The *Adamts20* gene was sequenced from *bt^9J^*/*bt^9J^* genomic DNA in order to identify the molecular lesion. A single C to T point mutation was identified at nucleotide 2451, which is predicted to cause a missense mutation (Leu761Phe) in the spacer domain of ADAMTS20. The leucine residue mutated in *bt^9J^* mice is present in both mouse and human *Adamts20* genes and is highly conserved among all *Adamts* mouse genes (present in 15 of the 19 genes). This suggests that Leu761 may be a critical residue for ADAMTS20 folding or for enzymatic function. We designed a Taqman^TM^ assay to genotype *bt^9J^* mice (see Materials and Methods). We determined that *bt^9J^* is the same strain as Mutant Mouse Resource Center (MMRC) strain #183 (Figure 1B). Although the missense mutations in *bt^9J^*, *bt^Mri1^*, and *bt* affect different domains of ADAMTS20, these and other *bt* alleles are recessive and exhibit very similar phenotypes, strongly suggesting they act as functional null alleles.

### Initial Specification and Migration of Melanoblasts Is not Disrupted in *bt^9J^/bt^9J^* Embryos

White spotting of mouse coats is typically caused by defective melanoblast development during embryogenesis. In order to elucidate when and how *Adamts20* affects melanoblast development, melanoblast distribution in *bt^9J^/bt^9J^* embryos was examined. We generated C57BL/6 mice containing a *Dct*-LacZ transgene, which marks specified melanoblasts, and crossed these onto a C57BL/6 *bt^9J^*/*bt^9J^* background [18],[32]. Since there are no phenotypes associated with *bt^9J^*/+ adult animals, this heterozygous genotype served as a control for this study.

**Figure 2:**
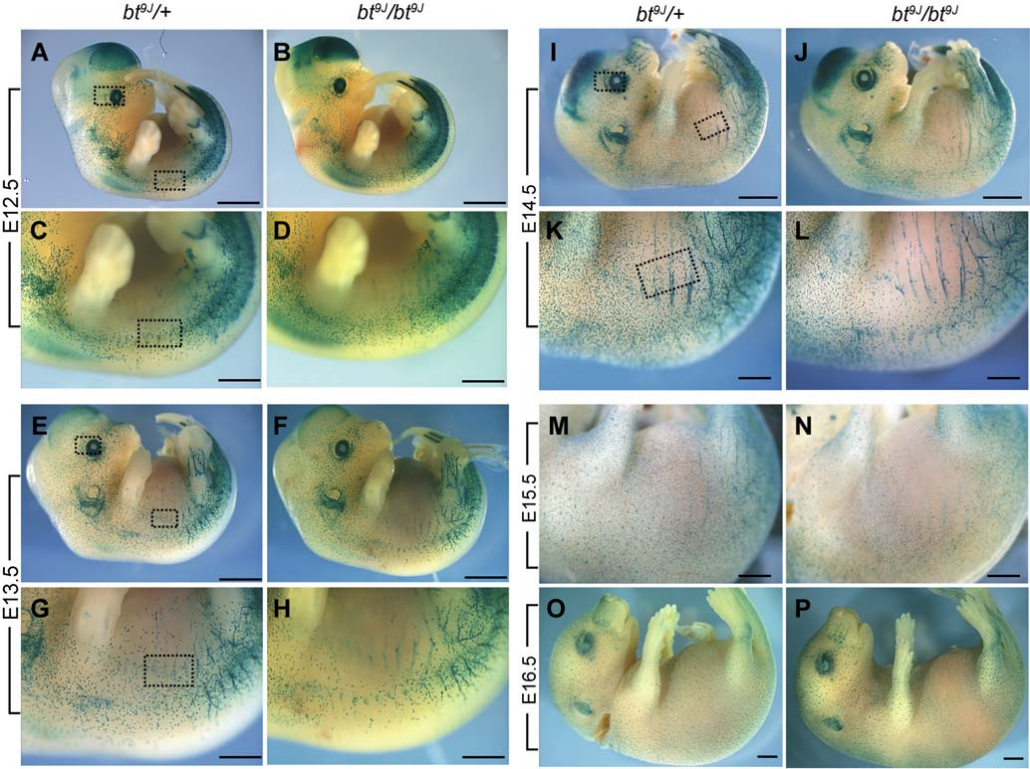
Melanoblast distribution is defective in whole mount E13.5 and older *bt^9J^/bt^9J^* embryos. (A–P) Images of β-galactosidase stained whole mount *bt^9J^/+;Dct-*LacZ (A,C,E,G,I,K,M,O) and *bt^9J^/bt^9J^;Dct-LacZ* embryos (B,D,F,H,J,L,N,P). Shown are E12.5 embryos (A,B), E12.5 trunks (C,D), E13.5 embryos (E,F), E13.5 trunks (G,H), E14.5 embryos (I, J), E14.5 trunks (K, L), E15.5 trunks (M,N) and E16.5 embryos (O,P). Each image is representative of at least 12 embryos. Boxes in A, C, E, G, I, K indicate regions in which melanoblasts were quantitated; the indicated regions were quantitated in both *bt^9J^/+;Dct-*LacZ and *bt^9J^/bt^9J^;Dct-LacZ* embryos (see Table 1). Scale bars are: (A,B,E,F,I,J,O,P) 2 mm, (C,D,G,H,K,L,M,N) 1 mm.

We compared melanoblast distribution in whole mount *bt^9J^*/+;*Dct*-LacZ and *bt^9J^*/*bt^9J^*;*Dct-*LacZ embryos at E11.5 through E16.5 (Figure S1 and Figure 2). In E11.5 and E12.5 control embryos, melanoblasts had completed their initial migration from the neural tube and were distributed evenly across the dorsal and lateral surfaces of the embryo (Figures S1 and Figure 2A and 2C). A similar distribution pattern in E11.5 and E12.5 *bt^9J^*/*bt^9J^* embryos was observed (Figures S1 and Figure 2B and 2D). Quantification of melanoblasts in the head and the presumptive belt region of the trunk of E12.5 whole mount embryos showed no difference in melanoblast number between control and *bt^9J^*/*bt^9J^* embryos (Table 1, boxes in Figure 2A and 2C). These results show that initial specification and migration of NC-derived melanoblasts is not disrupted in *bt^9J^*/*bt^9J^* animals.

**Table 1:** Quantitation of melanoblasts in whole mount embryos.

Age	Location	*bt^9J^/+*	*bt^9J^/bt^9J^*	P value
E12.5	Eye	162±85 (16)	190±48 (19)	0.13241
	Trunk	162±102 (16)	197±59 (19)	0.11472
E13.5	Eye	171±27 (10)	159±18 (10)	0.25806
	Trunk	105±19 (10)	42±14 (10)	0.0000002 **
E14.5	Eye	128±36 (8)	134±30 (10)	0.35728
	Trunk	220±69 (8)	56±23 (10)	0.000096 **

Shown are the average number of melanoblasts quantitated, standard deviations, and the number of samples in parentheses. P values are included with ** indicating highly significant values. The regions quantitated are represented in Figure 2 and described in the Materials and Methods.

### Melanoblast Distribution Is Reduced only in the Trunk of *bt^9J^/bt^9J^* Embryos

Since there was no defect in younger embryos, melanoblast distribution in *bt^9J^*/+; *Dct-*LacZ and *bt^9J^*/*bt^9J^*;*Dct-*LacZ E13.5-E16.5 embryos was compared (Figure 2E–2P). In E13.5 and E14.5 *bt^9J^*/+;*Dct-*LacZ embryos, the melanoblast population had expanded throughout the embryo and melanoblasts were distributed uniformly across dorsal and lateral surfaces of the trunk (Figure 2E, 2G, 2I, and 2K). In contrast, melanoblast distribution was specifically reduced in the trunk of E13.5 and E14.5 *bt^9J^*/*bt^9J^*;*Dct-*LacZ embryos (Figure 2F, 2H, 2J, and 2L). Quantitation of the head and trunk region of E13.5 and E14.5 *bt^9J^*/*bt^9J^*;*Dct-*LacZ embryos confirmed that the melanoblast defect was limited to the trunk (Table 1, boxes in Figure 2E, 2G, 2I, 2K) (p = 0.0000002 at E13.5, p = 0.000096 at E14.5). By E15.5 and E16.5, melanoblasts were distributed evenly on both dorsal and ventral surfaces of *bt^9J^*/+;*Dct-*LacZ embryos (Figure 2M and 2O). However *bt^9J^*/*bt^9J^*;*Dct-*LacZ embryos displayed a notable absence of melanoblasts on both surfaces in the region corresponding to the future belt (Figure 2N and 2P). Melanoblast distribution in the head and tail regions was similar between E16.5 *bt^9J^*/+;*Dct-*LacZ and *bt^9J^*/*bt^9J^*;*Dct-*LacZ embryos (Figure 2O and 2P). Close examination of the belt region in E16.5 *bt^9J^*/*bt^9J^*;*Dct-*LacZ embryos showed no accumulation of melanoblasts at the edges of the belt, as might be expected if migration into this region were impaired (Figure S2). Collectively, these analyses of the melanoblast distribution in *bt^9J^*/*bt^9J^* animals reveal the following: the phenotype is first apparent at E13.5, melanoblasts are reduced only in the trunk region (the location of the presumptive belt), and dorso-lateral melanoblast migration is not impaired.

### Melanoblasts Are Present in All Skin Compartments of *bt^9J^/bt^9J^* Embryos

**Figure 3:**
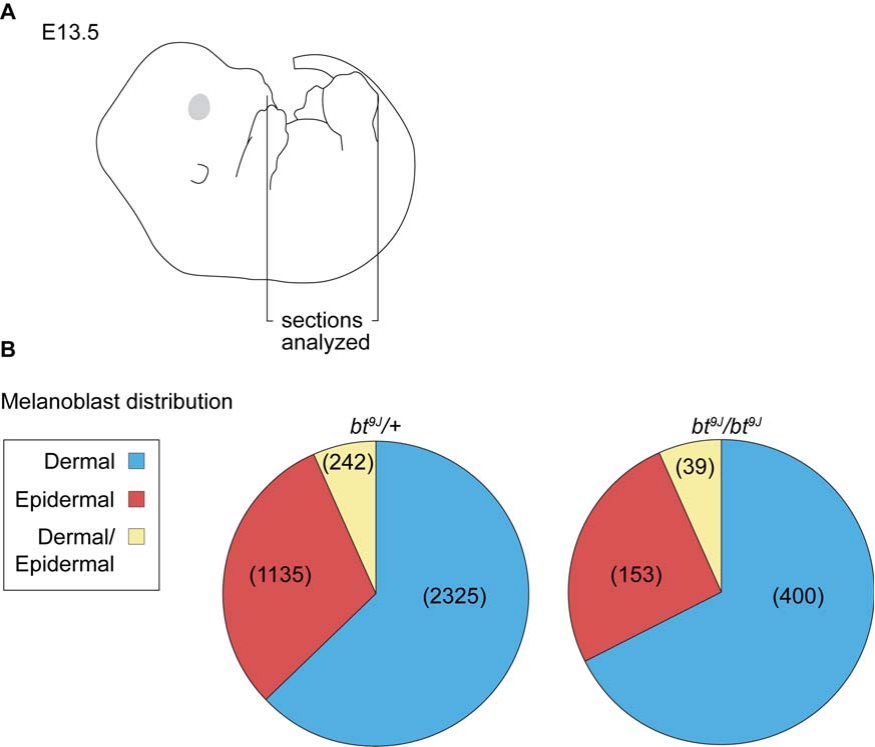
Melanoblast distribution in the skin layers of E13.5 *bt^9J^/bt^9J^* embryos is normal. (A) A cartoon representation of an E13.5 whole mount embryo showing the region between the fore- and hind-limbs from which 16 µm trunk sections were analyzed. (B) Pie charts representing the melanoblast distribution in the dermis (blue), epidermis (red), and dermal/epidermal border (yellow) from E13.5 *bt^9J^/+* (top) and *bt^9J^/bt^9J^* (bottom) sections with the total number of melanoblasts indicated in parentheses (n = 2 embryos, each genotype). There was no significant difference in melanoblast distribution between *bt^9J^/+* and *bt^9J^/bt^9J^* embryos (p = 0.056).

The melanoblast defect is first observed at E13.5, coincident with the timing of melanoblast migration from the dermis into the epidermis, and with generalized dermal expression of *Adamts20* [12],[15]. Therefore we hypothesized that *Adamts20* may be required for normal distribution of melanoblasts in dermal and epidermal compartments of the skin. To address this, melanoblasts were quantified in the dermis, epidermis, and dermal-epidermal border in E13.5 trunk sections (Figure 3A and Table 2) (see Materials and Methods). In control trunk sections (n = 178) we observed an average of 33.6 melanoblasts per section, whereas in *bt^9J^*/*bt^9J^*;*Dct-*LacZ sections (n = 179) there was an average two-fold reduction in melanoblast number (15.9 per section, p<0.0001) (Table 2). This finding is consistent with the analyses of whole mount embryos (see Table 1 and Figure 2E–2H). However, the relative distribution of melanoblasts within each of the skin layers in *bt^9J^*/*bt^9J^*;*Dct-*LacZ embryos did not differ significantly from *bt^9J^*/+;*Dct-*LacZ control embryos (p = 0.056) (Figure 3B). These results show that while melanoblast number is significantly reduced in the lumbar region of *bt^9J^*/*bt^9J^* embryos, melanoblast migration from the dermis into the epidermis is unaffected.

**Table 2:** Quantitation of Melanoblasts in sections through the head and trunk at E13.5.

Location	Genotype	Total sections analyzed	Average number Melanoblasts/section	Average % apoptotic Melanoblasts/section
Head	*bt^9J^/+*	40	110 (±22)	0.3 (±0.6)
	*bt^9J^/bt^9J^*	45	117 (±36) NS	1.2 (±2.2)**
Trunk	*bt^9J^/+*	178	33.6 (±20)	1.2 (±2.6)
	*bt^9J^/bt^9J^*	179	15.9 (±14)**	8.4 (±16.9)**

At least 3 embryos of each genotype were analyzed. For the head, only horizontal sections containing one or both eyes were included. For the trunk, horizontal sections corresponding to regions shown in Figure 3 were included. The standard deviations are included in parentheses. The results of statistical analyses comparing *bt^9J^/+* and *bt^9J^/bt^9J^* are included (NS = not significant and ** = p<0.0001, which is highly significant).

### *bt^9J^*/*bt^9J^* Embryos Exhibit Normal Melanoblast Proliferation but Defective Melanoblast Survival

**Figure 4:**
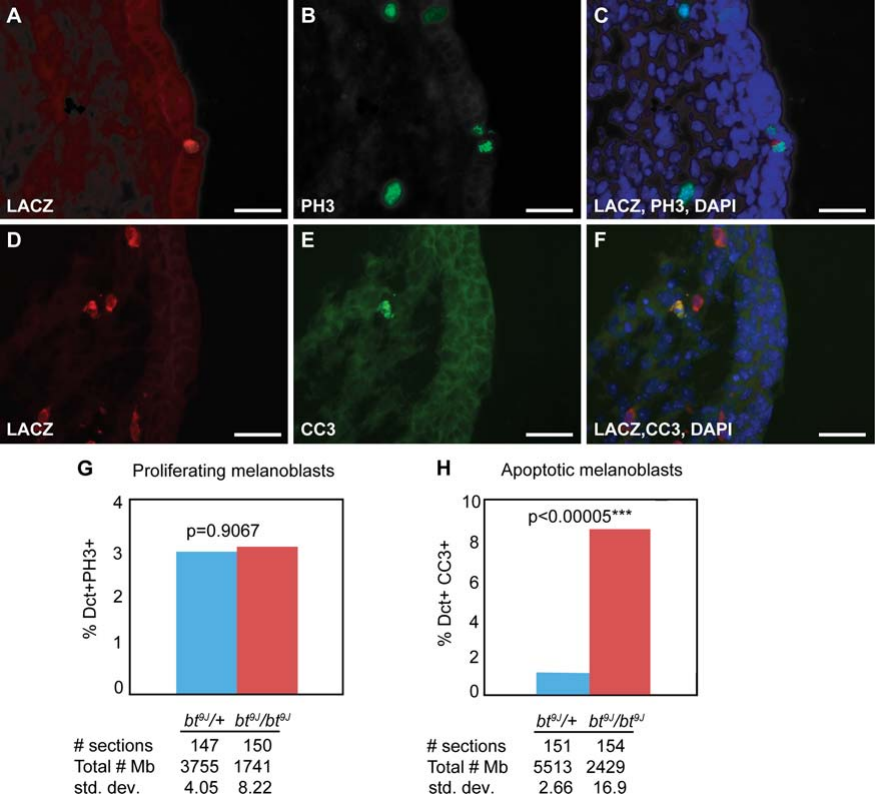
Melanoblast proliferation and apoptosis in *bt^9J^/+* and *bt^9J^/bt^9J^* embryos. (A–F) Representative immunofluorescence images depicting 16 µm trunk sections from *bt^9J^/bt^9J^* E13.5 embryos assayed for proliferation (A–C) and apoptosis (D–F). Proliferating melanoblasts were identified by staining sections for LacZ (cytoplasmic red, A) and PH3 (nuclear green, B), and their co-localization together with nuclear DAPI staining (blue, C). Apoptotic melanoblasts were identified by staining sections for LacZ (cytoplasmic red, D) and CC3 (cytoplasmic green, E), and their co-localization together with DAPI (blue, F). (G–H) The Y axis (%Dct+PH3+ and %Dct+CC3) indicates the average percentage of total melanoblasts per section that are PH3 positive (G) or CC3 positive (H), for *bt^9J^/+* (blue) and *bt^9J^/bt^9J^* (red) E13.5 embryos (n = 5 embryos for each genotype). The total number of sections and melanoblasts analyzed are listed beneath each graph, as well as the standard deviations. Asterisks indicate highly significant P values. The scale bars are: (A–F) 0.5 µM.

At E13.5, when the defect in melanoblast development in *bt^9J^*/*bt^9J^* embryos is first apparent, melanoblasts are undergoing dramatic increases in cell proliferation [18]. To assess if the *bt* phenotype is caused by reduced melanoblast proliferation in the trunk region of *bt^9J^*/*bt^9J^* embryos, the mitotic index of melanoblasts at E13.5 was examined in *bt^9J^*/+;*Dct-*LacZ and *bt^9J^*/*bt^9J^*;*Dct-*LacZ trunk sections (Figures 3A and 4). Melanoblasts undergoing mitosis were identified by co-expression of β-galactosidase and phospho-histoneH3 (PH3) (Figure 4A–4C). The average percentage of dividing melanoblasts in control and *bt^9J^*/*bt^9J^* sections was not significantly different (2.94% and 3.03% respectively, p = 0.9067) (Figure 4G), suggesting that the melanoblast reduction in *bt^9J^*/*bt^9J^* embryos is not a consequence of abnormal proliferation.

The reduced number of melanoblasts in the trunk region of E13.5 *bt^9J^*/*bt^9J^*;*Dct-*LacZ embryos could also be due to a failure of melanoblasts to survive [33]. To examine this, the apoptotic index of melanoblasts was quantitated from cells co-expressing β-galactosidase and cleaved-caspase3 (CC3) (Figures 3A and 4D–4F). The average percentage of apoptotic melanoblasts was significantly increased in *bt^9J^*/*bt^9J^*;*Dct-*LacZ sections relative to *bt^9J^*/*+*;*Dct-*LacZ sections (compare 8.4% to 1.2%, p<0.00005) (Figure 4H, Table 2). Apoptotic melanoblasts were apparent in both dorsal and ventral skin in control and mutant embryos. In addition, no significant differences were seen in the distribution of apoptotic, CC3+melanoblasts in the three skin compartments, as follows: dermal (*bt^9J^*/*bt^9J^* = 77.8%; control = 82.4%), epidermal (*bt^9J^*/*bt^9J^* = 14.8%; control = 5.8%), and dermal/epidermal (*bt^9J^*/*bt^9J^* = 7.4%; control = 11.85%), (p = 0.6133). These results show that *Adamts20* is required for melanoblast survival in all cell layers of the trunk at E13.5.

*Adamts20* is expressed along the length of the embryo, yet the spotting phenotype in adult mice is evident only in the lumbar region. Therefore we examined melanoblast number and melanoblast apoptosis in a region outside of where the belt occurs, in the head (Table 2). In normal embryos, the density of melanoblasts is not the same in all regions, as previously described [34]–[37]. As expected, quantitation of melanoblasts in control embryos demonstrated that there were over three-fold more total melanoblasts in the head region than in the trunk (compare 110 versus 33.6, p = 0.0001). There was no difference in melanoblast number in the head of control and *bt^9J^*/*bt^9J^*;*Dct-*LacZ embryos (Table 2), similar to what was seen in whole mount analyses.

The apoptotic index of melanoblasts varied in different regions of control embryos as well as between control and mutant embryos (Table 2). In control embryos, melanoblast apoptosis was lower in the head than in the trunk. Comparing control and *bt^9J^*/*bt^9J^* embryos, we found that apoptosis was increased in the head although to a lesser extent than observed in the trunk. This result indicates that *Adamts20* is required for melanoblast survival throughout the embryo even though it does not result in white spotting in the head.

### Melanoblasts in *bt^9J^*/*bt^9J^* Skin Fail To Respond to sKitl

**Figure 5:**
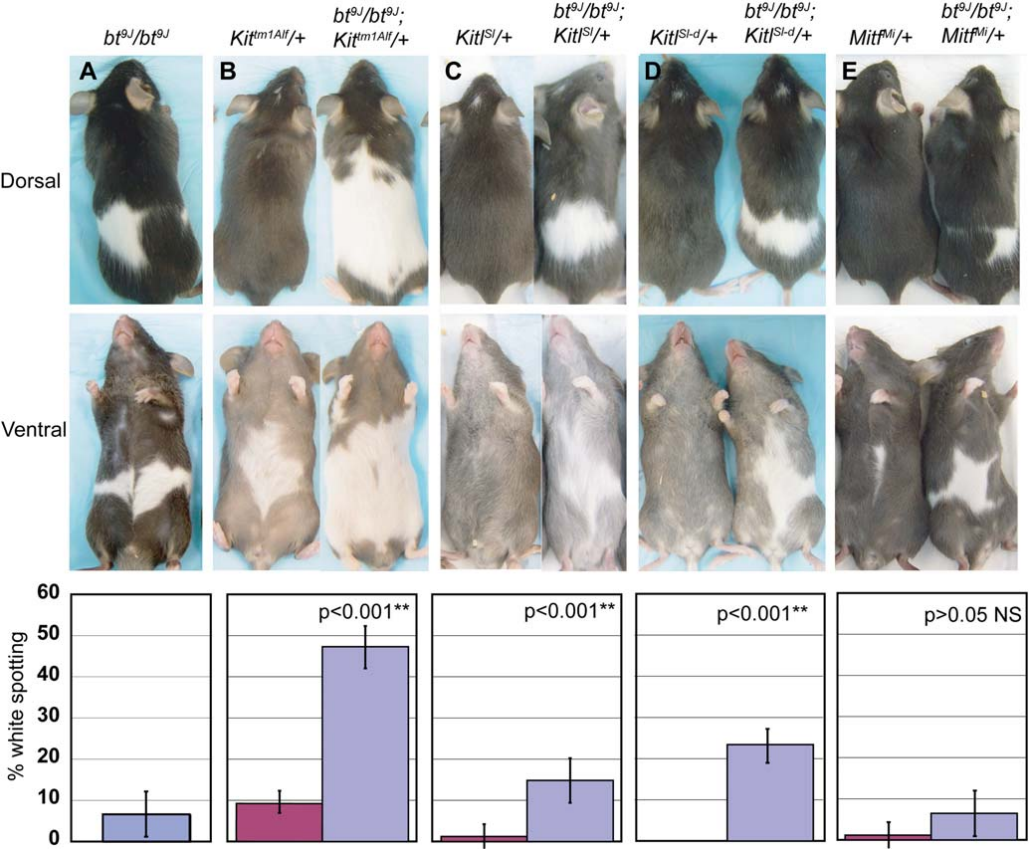
*bt^9J^/bt^9J^* animals exhibit synergistic spotting with heterozygous mutations in the *Kit* pathway. (A–E) Shown are representative dorsal and ventral images of mice (top and middle row, respectively). The genotypes and number of animals (n) analyzed were: (A) *bt^9J^/bt^9J^* (n = 31), (B) *Kit^tm1Alf^/+* (n = 14) and *bt^9J^/bt^9J^;Kit^tm1Alf^/+* (n = 15), (C) *Kitl^Sl^*/+ (n = 24) and *bt^9J^/bt^9J^*;*Kitl^Sl^*/+ (n = 17), (D) *Kitl^Sl-d^*/+ (n = 6) and *bt^9J^/bt^9J^*;*Kitl^Sl-d^*/+(n = 6), and (E) *Mitf^Mi^*/+ (n = 8), and *bt^9J^/bt^9J^*;*Mitf^Mi^*/+ (n = 13). Below are graphs showing quantitation of the average proportion of white spotting with standard deviations. P values represent comparisons of spotting between compound mutants and *bt^9J^/bt^9J^* animals, and between compound mutants and *Kit* mutants. Asterisks indicate highly significant values.

Melanoblast survival is dependent upon Kit activation [20],[38] by Kit ligand (Kitl), which is expressed in the dermis in a similar temporal and spatial pattern to *Adamts20* [12], [21], [24]–[26]. We hypothesized that *Adamts20* regulates melanoblast survival through modulation of Kit signaling, and assessed the effects of altered Kit signaling upon the extent of white spotting in *bt^9J^*/*bt^9J^* animals harboring various mutant alleles of *Kit* (MGI: 96677) or *Kitl* (MGI: 96974). *Kit^tm1Alf^*/+ mice contain a null mutation in *Kit* and have primarily ventral spotting [39]–[41]. As depicted in Figure 5, *bt^9J^*/*bt^9J^*;*Kit^tm1Alf^*/+ mice exhibited dramatically wider dorsal and ventral belts in comparison to *bt^9J^*/*bt^9J^* mice or *Kit^tm1Alf^*/+ mice alone (Figure 5A and 5B). The increased spotting was a synergistic effect rather than an additive one (compare 46.9% of dorsal and ventral surfaces in *bt^9J^*/*bt^9J^*;*Kit^tm1Alf^*/+ with 11.2% and 9.1% in *Kit^tm1Alf^*/+ and *bt^9J^*/*bt^9J^*, respectively). Synergistic increases in spotting were also observed in *bt^9J^*/*bt^9J^* mice carrying mutant alleles of *Kitl*. Heterozygosity for either *Kitl^Sl^*, a null allele, or *Kitl^Sl-d^*, a deletion that generates short soluble Kitl but not membrane–bound Kitl, combined with homozygosity for *bt^9J^* resulted in synergistically increased spotting (Figure 5C and 5D) [42],[43]. In contrast, *bt^9J^*/*bt^9J^* mice carrying a mutation in *Mitf* (*bt^9J^*/*bt^9J^;Mitf^Mi^/+*) (MGI: 104554) exhibited no synergistic spotting (Figure 5E). These results show that decreasing *Kit* signaling exacerbates the *bt^9J^*/*bt^9J^* phenotype.

Heterozygous *Kit^W-v^* mutations reduce melanoblast number beginning at E10.5 [18], which is three days prior to the onset of the melanoblast defect in *bt^9J^*/*bt^9J^*. To determine if decreasing Kit signaling in *bt^9J^*/*bt^9J^* embryos would lead to an earlier defect in melanoblast development than seen in *bt^9J^*/*bt^9J^* alone, we examined E12.5 *bt^9J^*/*+*;*Kit^tm1Alf^*/+ and *bt^9J^*/*bt^9J^*;*Kit^tm1Alf^*/+ embryos (Figure S3). Since the *Kit^tm1Alf^* mutation is a *LacZ* knock-in at the *Kit* locus, we monitored melanoblasts using β-galactosidase staining. There was no detectable difference in *Kit*-positive melanoblast distribution between *bt^9J^*/*+*;*Kit^tm1Alf^*/+ and *bt^9J^*/*bt^9J^*;*Kit^tm1Alf^*/+ embryos at E12.5. These results demonstrate that the synergistic defect in melanoblast development does not precede the onset of the *bt* phenotype at E13.5.

**Figure 6:**
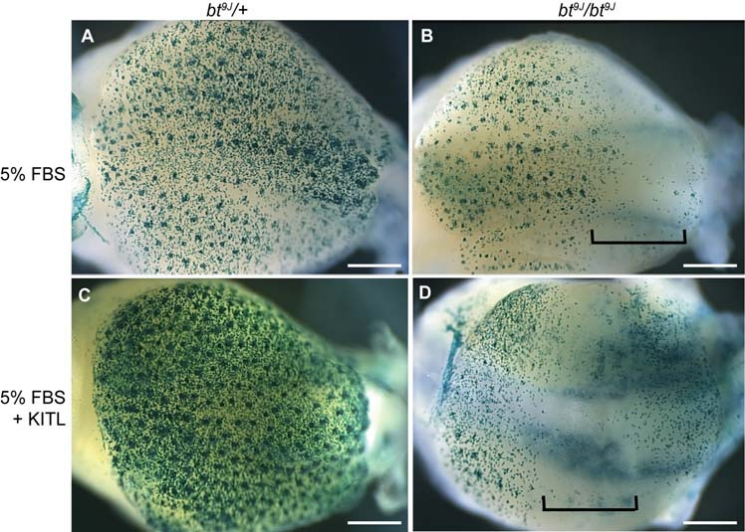
Melanoblasts in *bt^9J^/bt^9J^* skin do not respond to soluble Kitl. (A–D) Images of β-galactosidase stained skin cultures grown from E13.5 *bt^9J^/+;Dct*-LacZ (A,C) or *bt^9J^/bt^9J^;Dct*-LacZ (B,D) embryos, and treated with either 5% FBS (A,B) or 5% FBS+Kitl (C,D). Brackets indicate the presumptive belt regions in *bt^9J^/bt^9J^* skin. The scale bars are: (A–D) 0.5 mm.

The synergistic interaction studies indicate that Kit signaling may be disrupted in *Adamts20* mutant animals. To test if melanoblasts in *bt^9J^*/*bt^9J^* embryos could respond to sKitl we used an *ex vivo* embryonic skin explant assay using dorsal trunk skin from E13.5 embryos [44]–[46]. Observation of *bt^9J^*/*+*;*Dct-*LacZ and *bt^9J^*/*bt^9J^*;*Dct-*LacZ skin after four days in culture demonstrated that *ex vivo* explant culture conditions could recapitulate both normal melanoblast colonization and the *bt* defect (Figure 6). Melanoblasts in the control skin entered hair follicles and were distributed evenly across the explant (n = 5) (Figure 6A). While some melanoblasts survived and migrated into hair follicles in the *bt^9J^*/*bt^9J^*;*Dct-*LacZ skin, in a large domain of the explant the melanoblasts were reduced (n = 5) (Figure 6B), similar to the phenotype of whole mount E16.5 *bt^9J^*/*bt^9J^*;*Dct-*LacZ embryos (Figure 2O and 2P).

Previous studies showed that melanoblasts within embryonic skin cultures respond to soluble Kitl (sKitl) [44] and that sKitl promotes melanoblast survival in NC cultures [38]. ADAMTS20 could be required for proteolytic cleavage of Kit or Kitl, or for modifying additional molecules necessary for Kit signaling. To test if Kit signaling was defective in *bt^9J^*/*bt^9J^* embryos, we asked whether melanoblasts in *bt^9J^*/*bt^9J^* embryos could respond to sKitl and whether sKitl could rescue the *bt* phenotype. Similar to studies by Jordan et. al, an increase in melanoblast number and colonization of hair follicles was observed in control skin exposed to sKitl as compared to FBS alone (Figure 6C, n = 5). In contrast, exposure of *bt^9J^*/*bt^9J^*;*Dct-*LacZ skin (n = 7) to sKitl did not restore melanoblasts to the presumptive belt (Figure 6D). Interestingly, melanoblasts at the rostral and caudal regions of the trunk did not increase in number, even though these are outside of the expected belt. These results show that in the absence of *Adamts20,* melanoblasts are unable to respond to sKitl, indicating that Kit signaling is disrupted in *bt^9J^*/*bt^9J^* mutants. Furthermore, since sKitl could not rescue the *bt* phenotype, it suggests that the melanoblast defect in *bt^9J^/bt^9J^* animals is not caused by defective cleavage of Kitl.

### ADAMTS20 Cleaves Versican *in vitro* and Is Necessary for Versican Cleavage *in vivo*

We explored the possibility that *Adamts20* could regulate melanoblast survival through additional pathways. One of the best-understood activities of ADAMTS proteases is processing of various ECM substrates. Of note, ADAMTS20 is most closely related phylogenetically to ADAMTS metalloproteases that specifically process chondroitin sulfate proteoglycans (CSPGs) [1],[2],[47]. Although no substrates for ADAMTS20 have been identified, the CSPG versican is enriched in the skin and implicated in NC development, making it an excellent candidate substrate of ADAMTS20 [48]–[52].

We examined *Versican* and *Adamts20* expression by whole mount *in situ* hybridization in wild-type embryos. At E12.5, when melanoblasts are widely distributed in the trunk, both *Versican* and *Adamts20* are expressed broadly across the trunk, overlapping in many regions of the skin and the developing mammary ducts (data not shown). Since both versican and ADAMTS20 are secreted proteins, the expression patterns of their RNAs relative to each other and to melanoblasts support the possibility of a functional relationship *in vivo*.

**Figure 7:**
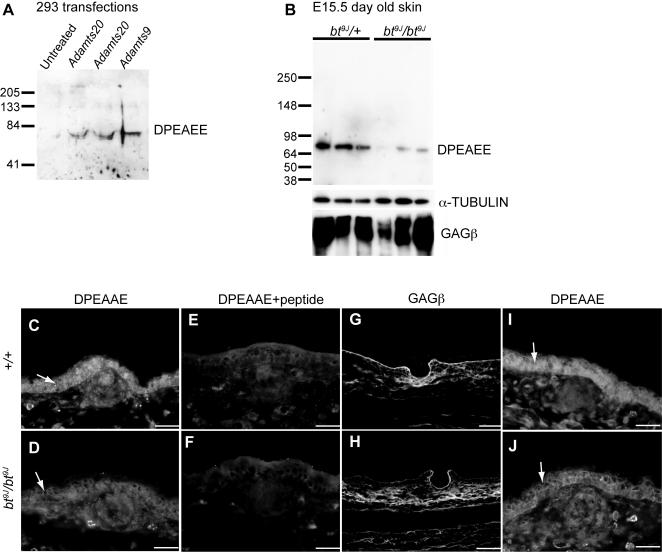
ADAMTS20 cleaves versican *in vitro* and *in vivo*. (A,B) Western analyses probed with anti-DPEAEE that recognizes the 70 KDa versican cleavage product. (A) Protein extracts from 293 cells transfected with vector alone, *Adamts20* (2 experiments) and *Adamts9,* and incubated with versican. (B) Protein extracts of E15.5 day old skin from six different *bt^9J^/+* and *bt^9J^/bt^9J^* embryos. Anti-α-Tubulin serves as a loading control and anti-GAG βindicates levels of total versican. (C–J) Immunofluorescence images of *+/+* (C,E,G, I) or *bt^9J^/bt^9J^* (D,F,H,J) E15.5 10 µm trunk (C–H) and forelimb (I,J) sections stained with anti-DPEAAE (C,D,I,J), anti-DPEAAE and DPEAEE peptide (E,F), and anti-GAG β (G,H). The anti-DPEAEE staining in the proliferating layer of the epidermis (indicated by arrows) is reduced dramatically in trunk sections and reduced to a lesser extent in forelimb sections from *bt^9J^/bt^9J^* embryos. The scale bars are: (C–F,I,J) 50 µM, (G,H) 100 µM.

We assessed the ability of ADAMTS20 to process versican by expressing ADAMTS20 in 293 cells, and subsequently incubating these cells with versican (Figure 7A). *Versican* is alternatively spliced to generate four isoforms, with *VersicanV1* being the dominant variant in adult skin [53]–[56]. Versican cleavage was examined using anti-DPEAEE antibody, which specifically recognizes the neo-epitope generated by ADAMTS cleavage of versicanV1 (70 KDa) [57]. While secreted media from 293 cells alone exhibited little to no cleavage of versican, versican processing was evident in secreted media from 293 cells expressing *Adamts20* (Figure 7A). As a positive control, cleaved versican was also evident in secreted media from cells expressing *Adamts9*, which has previously been shown to process versican [47]. These results show that *in vitro,* versican is a substrate for cleavage by ADAMTS20.

Analysis of skin extracts from the dorsal trunk of E15.5 *bt^9J^*/*+* and *bt^9J^*/*bt^9J^* embryos showed ADAMTS20 is also necessary for versican cleavage *in vivo* (Figure 7B). The levels of the cleaved 70 KDa band were reduced in *bt^9J^*/*bt^9J^* extracts compared to control extracts (n =  4 experiments). We also observed a reduction in levels of a 220 KDa band corresponding to the V0 isoform (data not shown). In contrast, there was no alteration in total versican, which was assessed using a GAG β antibody that recognizes the GAG β domains of full-length versicanV0 and V1 (Figure 7B). Collectively, these results show that ADAMTS20 cleaves versican *in vitro* and that it is required for versican cleavage in skin *in vivo*.

Immunofluorescence was performed to evaluate the spatial pattern of versican cleavage in embryonic skin. Versican expression was assessed on trunk sections of E15.5 C57BL/6 (*+/+*) and *bt^9J^*/*bt^9J^* embryos (Figure 7C–7H). In *+/+* sections, cleaved versican was evident in the dermal mesenchyme and was enriched in the proliferating basal layer of the epidermis (arrow in Figure 7C) but was excluded from the outermost layer of the epidermis, in a similar pattern to that of intact versican in adult human skin [56]. In contrast to *+/+* sections, *bt^9J^*/*bt^9J^* sections displayed a dramatic reduction in cleaved versican in the epidermis (Figure 7D). Inclusion of the DPEAEE peptide in the staining procedure demonstrated the specificity of the antibody for this peptide (Figure 7E and 7F). Total versican expression was similar in both *+/+* sections and *bt^9J^*/*bt^9J^* sections, with high expression in the basement membrane, dermis, and dermal condensations, but lower levels in the epidermis (Figure 7G and 7H). Outside of the belt region, at the level of the forelimb, the levels of cleaved versican in *bt^9J^*/*bt^9J^* sections were reduced relative to wild-type, but not to the same extent as seen in the trunk (Figure 7I and 7J). Together these results show that in *bt^9J^*/*bt^9J^* embryos, levels of processed versican, but not total versican, are reduced in the skin of the embryonic trunk, and show that ADAMTS20 is necessary for versican remodeling during melanoblast development.

### *Adamts9* and *Adamts20* Act Cooperatively To Regulate Melanoblast Development

Although *Adamts20* is expressed across the length of the embryo [12], only the lumbar region of the trunk in *bt^9J^*/*bt^9J^* mutants shows dramatic reduction of melanoblasts and white spotting. Given that ADAMTS20 belongs to a large family of closely related metalloproteases, we reasoned that partial redundancy with other metalloproteases might account for the higher proportion of melanoblasts surviving outside of the lumbar region. The two closest *Adamts20* homologs, *Adamts5* (GeneID: 23794) and *Adamts9,* are both expressed in the embryonic skin along the length of the embryo and are both versicanases, making them excellent candidates for regulating melanoblast development [47],[58],[59](Bon-Hun Koo and S.S. Apte, unpublished data). To test if these metalloprotease genes were functionally redundant with *Adamts20*, we evaluated the extent of white spotting in mutants lacking *Adamts20* and either *Adamts9 (Adamts9^ko^*) or *Adamts5 (Adamts5^ko^)*.

**Figure 8:**
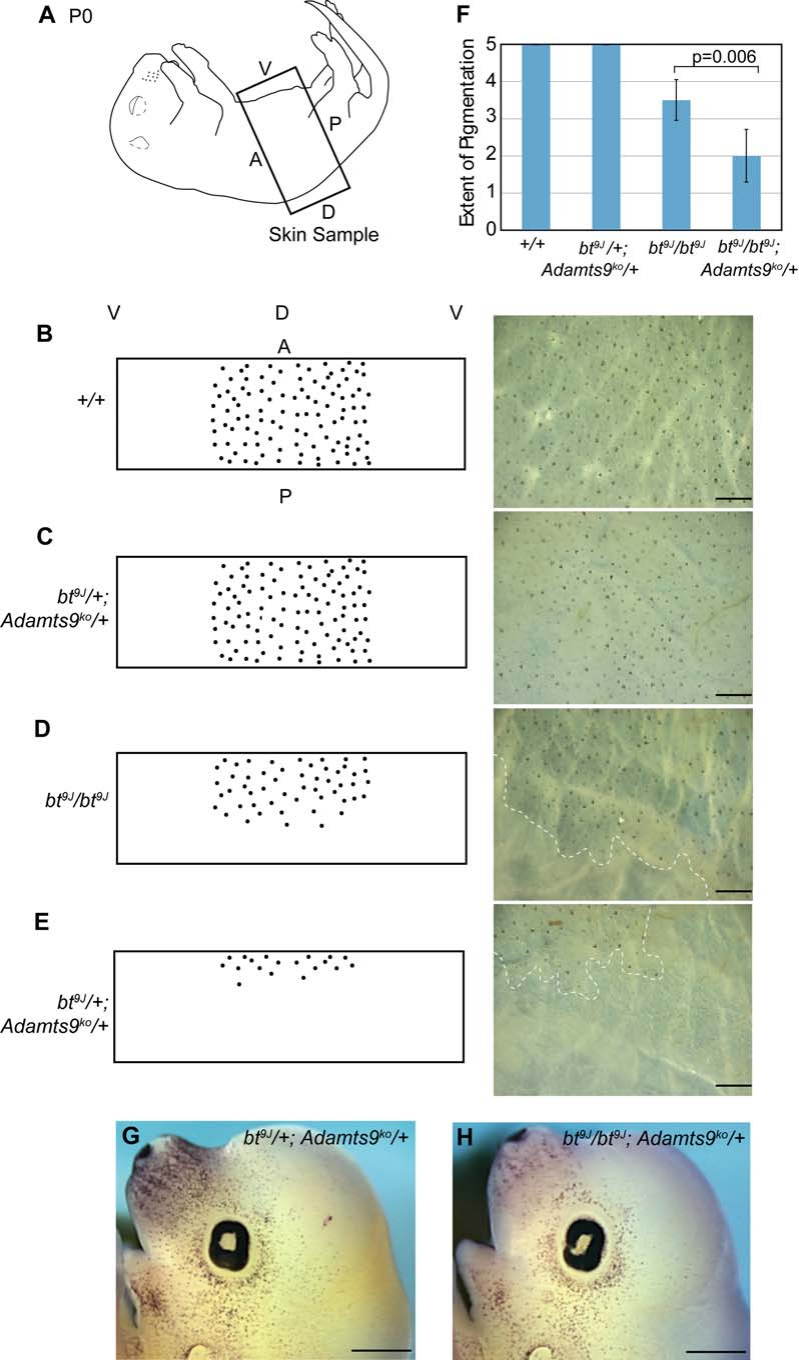
*Adamts9* and *Adamts20* act redundantly to regulate pigmentation. (A) A schematic image of a newborn pup. Skin samples (indicated by the box) were taken from the trunk region around the circumference of the animal and were flattened with a single cut on the ventral side. (B–E) Shown on the left are cartoon representations of the pigmentation patterns and on the right are high magnification images of skin samples from (B) +/+ (n = 4), (C) *bt^9J^/+; Adamts9^ko^/+*(n = 4), (D) *bt^9J^/bt^9J^* (n = 6), and (E) *bt^9J^/bt^9J^; Adamts9^ko^/+*(n = 5) animals. Dots indicate pigmented hair follicles. Labels for orientation of the samples are as follows: V (ventral), D (dorsal), A (anterior), and P (posterior). Images were taken by focusing on 1/3 of the anterior-posterior length of the skin sample. (F) A graph depicting the relative score of pigmentation as described in the Materials and Methods. The difference between *bt^9J^*/*bt^9J^* and *bt^9J^*/*bt^9J^*; *Adamts9^ko^/+* samples was significantly different (p = 0.006). (G and H) Head images of *in situ* hybridization with the melanoblast marker, *Pmel17*, on whole mount E13.5 *bt^9J^/+; Adamts9^ko^/+*(n = 2) (G) and *bt^9J^/bt^9J^; Adamts9^ko^/+*(n = 2) (H) embryos. Note there are fewer melanoblasts all over the head and particularly in the snout of *bt^9J^/bt^9J^; Adamts9^ko^/+*embryos. Scale bars = 0.5 mm.

*Adamts9^ko^* homozygotes are lethal prior to gastrulation (H. Enomoto and S. Apte, submitted) therefore we used *Adamts9^ko^* heterozygotes to examine a functional overlap between *Adamts9* and *20*. While mice heterozygous for mutation of *Adamts20* and *9* are viable, *bt^9J^*/*bt^9J^;Adamts9^ko^/+* animals die shortly after birth (H. Enomoto and S. Apte, submitted). We examined pigmentation in newborn animals that were wild-type, *bt^9J^*/*+;Adamts9^ko^/+, bt^9J^*/*bt^9J^*, or *bt^9J^*/*bt^9J^;Adamts9^ko^/+*. Skin pelts containing dorsal and ventral surfaces and spanning the entire trunk region were removed from newborn pups (Figure 8A). Although not externally obvious until 4–5 days after birth, melanoblasts can be seen as clusters of pigmented cells on the dermal side of the P0 epidermis. In all skin samples, pigmentation was apparent primarily on dorsal surfaces as shown by schematic representations in Figure 8B–8E. In the skin from *+/+* and *bt^9J^*/*+;Adamts9^ko^/+* animals, pigmentation was distributed uniformly between anterior and posterior boundaries of the skin samples (n = 4 each) (Figures 8B and 8C). In the *bt^9J^*/*bt^9J^* skin samples (n = 6), a partial de-pigmentation was observed on about 50% of the anterior-posterior length, consistent with the likely boundaries of “belted” and “non-belted” regions (Figure 8D). However, in the *bt^9J^*/*bt^9J^;Adamts9^ko^/+* skin samples (n = 5), de-pigmentation was apparent on 75% of the anterior-posterior length (Figure 8E). Our analyses showed a statistically significant increase in the severity of de-pigmentation in *bt^9J^*/*bt^9J^;Adamts9^ko^/+* skin compared to that seen in *bt^9J^*/*bt^9J^* skin (Figure 8F). In contrast, the pigmentation phenotype in *bt^Bei1^/bt^Bei1^;Adamts5^ko^/Adamts5^ko^* mice was similar to that of *bt^Bei1^/bt^Bei1^* animals indicating that loss of *Adamts5* does not exacerbate the *bt^9J^*/*bt^9J^* phenotype and that unlike *Adamts9, Adamts5* does not participate in melanoblast development (D. McCullough and S. Apte, unpublished data).

In order to assess melanoblast development in *bt^9J^*/*+;Adamts9^ko^/+*and *bt^9J^*/*bt^9J^;Adamts9^ko^/+* mice, E13.5 embryos were examined using *in situ* hybridization with *Pmel17* (GeneID: 20431), a marker specific for melanoblasts. Similar to what was observed in newborn skin, there was a significant reduction in melanoblasts in the trunk region of *bt^9J^*/*bt^9J^;Adamts9^ko^/+* embryos compared to *bt^9J^*/*+;Adamts9^ko^/+* embryos (data not shown). Importantly, there was also a significant reduction of melanoblasts in regions outside of the trunk (Figure 8G and 8H). These results indicate that *Adamts9* and *Adamts20* are partially redundant during melanoblast development not only in the trunk but in the head as well.

## Discussion

Analysis of mouse coat color mutants has yielded mechanistic insights into normal developmental pathways as well as human disease processes. In this paper we explored how mutation of *Adamts20,* a secreted metalloprotease that is not expressed in melanoblasts, can disrupt melanoblast development and cause white spotting. The expression pattern of *Adamts20*, along with studies performed on the *C.elegans* ortholog *Gon-1*, initially suggested that *Adamts20* regulated melanoblast migration [12], [27]–[29]. However, rather than this predicted requirement for melanoblast migration, we have identified an unexpected role for *Adamts20* in melanoblast survival. Our results have implications not only for understanding mechanisms of melanoblast development and pigmentation-associated diseases but also for the biological functions of secreted metalloproteases.

### Mutation of *Adamts20* Increases Melanoblast Apoptosis

The most striking alteration observed in *bt^9J^/bt^9J^* embryos was a reduction in trunk melanoblasts beginning at E13.5. Early events of melanoblast specification, migration, proliferation and survival were not affected, as the number and distribution of *Dct*-LacZ-positive melanoblasts was similar between control and *bt^9J^/bt^9J^* embryos up to E12.5. This result contrasts many melanoblast mutants including *Kit*, *Mitf*, *Pax3* (MGI: 97487), *Ednrb* (MGI: 102720), and *Sox10* (MGI: 98358), in which melanoblast defects can be observed as early as E10.5-E11.5 [15].

Multiple lines of evidence also indicate that later stages of melanoblast migration were not defective in *bt^9J^/bt^9J^* embryos. *Dct*-LacZ-positive cells did not accumulate dorsally or in the dermis, suggesting that melanoblast migration along the dorso-lateral pathway and between skin layers was normal. It is also unlikely that melanoblasts undergo cell death as a result of or subsequent to failed migration, as apoptotic melanoblasts were apparent in both dorsal and ventral regions of mutant embryos and in similar proportions within dermal and epidermal compartments. Our results also exclude a defect in the second wave of melanoblast migration [37],[60], since melanoblasts did not accumulate at the belt edges in E16.5 embryos. Instead, our results show that reduction of melanoblasts is due to the seven-fold increase in apoptosis observed in the trunk region of *Adamts20* mutant embryos.

### Why Do Mutations in *Adamts20* Cause a Belt?

We propose that the white belt occurs in *Adamts20* mutants for a combination of three factors: uneven densities of melanoblasts along the length of normal embryos, regional differences in apoptosis, and functional redundancy with other metalloproteases.

In wild-type animals there are fewer embryonic melanoblasts in the trunk relative to other areas of the body, as shown in this study and by others [17], [34]–37. Related to this, many of the coat color mutants exhibit spotting in the lumbar level of the trunk suggesting that melanoblasts in this region are particularly sensitive to genetic perturbation, perhaps due to reduced numbers. This suggests that if melanoblast numbers were further reduced along the trunk in *Adamts20* mutants, the resulting belt would be wider. Indeed this was the case since genetic interactions of *bt^9J^/bt^9J^* with *Kit* mutations caused a wider belt as opposed to increased spotting in the head.

Second, *Adamts20* is required for melanoblast survival throughout the embryo, consistent with the expression pattern of *Adamts20* along the length of the embryo. In *bt^9J^/bt^9J^* embryos, we found significant increases in apoptosis in both head and trunk regions. Importantly, the proportion of melanoblasts undergoing apoptosis was much more pronounced in the trunk than in the head. Thus a combination of increased apoptosis and lower initial melanoblast number in the trunk region of a *bt^9J^/bt^9J^* mutant depletes melanoblast numbers to levels at which it cannot generate adequate pigmentation.

Third, *Adamts20* mutant animals have limited white spotting due to functional redundancy with other metalloproteases, including *Adamts9.* In a *bt^9J^/bt^9J^* background, loss of a single copy of *Adamts9* results in reduced melanoblast numbers in the trunk as well as in the head. Thus, in *bt^9J^/bt^9J^* mice, *Adamts9* can compensate for *Adamts20* deficiency in regions of higher melanoblast number such as the head. The early embryonic lethality of *Adamts9* ^ko^/*Adamts9* ^ko^ mice makes it impossible to currently assess the absolute requirement of *Adamts9* in pigmentation. However, the widespread expression of *Adamts9* in the skin paired with the dramatic defect in melanoblast development in *bt^9J^*/*bt^9J^;Adamts9^ko^/+* animals predict an even greater pigmentation defect in a homozygous background. These future analyses will depend upon the generation of a conditional *Adamts9* knock-out in the skin.

### *Adamts20* Modulates Kit Signaling

Our results suggest that *Adamts20* is required for melanoblast survival at least in part through modulating *Kit* signaling. We show that *bt^9J^*/*bt^9J^*;*Kit^tm1Alf^*/*+* mutants exhibit synergistic spotting, and melanoblasts in *bt^9J^/bt^9J^* skin cultures are unable to respond to sKitl. This mechanism is consistent with the requirement of *Kit* signaling for melanoblast survival as well as the overlapping expression of *Adamts20*, *Kit* and *Kitl* in the dermis [12], [17], [20], [24]–[26],[61].

Kit signaling is essential for several events in melanoblast development beginning at E10.5 and including both migration and survival. Yet our findings indicate that mutation of *Adamts20* modulates only a subset of *Kit* functions, namely survival, and only during a defined window of time, around E13.5, rather than throughout development. This is evidenced by the fact that *bt^9J^/bt^9J^* embryos do not show altered melanoblast development prior to E13.5, such as seen in *Kit/+* embryos [17],[18]. In addition, *Kit* heterozygous mutations do not exacerbate the onset of the melanoblast defect in E12.5 *bt^9J^/bt^9J^* embryos, as would be expected if *Adamts20* were required earlier. The stage at which *Adamts20* disrupts melanoblast development may also explain why *bt* mice do not exhibit extensive ventral spotting like other white spotted mutants that impede earlier events in development.

Given these observations, it is notable that restricted disruption of *Kit*, during the embryonic period associated with onset of the *bt^9J^/bt^9J^* phenotype, results in mice with spotting phenotypes similar to *bt* [23],[62]. Pregnant mice injected with neutralizing Kit antibodies between E10.5 and E13.0 give birth to mice exhibiting white belts. In contrast, injection of these Kit antibodies into pregnant animals prior to E10.5 and after E13.0 results in mostly unpigmented mice.

A white belt/band is also evident in *Kit^W-sh^/+, Kit^W-57J^/Kit^W-57J^, and Kit^W-bd^/+* mice that carry mutations in *Kit* regulatory sequences and cause altered *Kit* expression patterns [35],[63],[64]. In addition, Kimura and colleagues describe belted phenotypes in *Kit^tm1Ber^* and *Kit^tm2Ber^* mice containing targeted mutations of Kit at tyrosine residues 567 and 569, amino acids essential for proper phosphorylation and downstream signaling [65]. It is interesting that disruption of these specific amino acids results in spotting similar to that seen in *belted* mice, and we speculate that *Adamts20* may be required for efficient Kit phosphorylation at these sites in melanoblasts *in vivo*.

There are several possible mechanisms by which ADAMTS20 could regulate Kit signaling. ADAMTS20 could be required directly for cleavage of either Kit and/or Kitl to produce sKitl. For example, the ADAM family member ADAM17 (GeneID: 11491) cleaves Kit *in vitro*, and ADAM17, 19 (GeneID: 8728) and 33 (GeneID: 110751) cleave Kitl [66]–[69]. However, sKitl did not rescue the melanoblast defect in *bt^9J^/bt^9J^* skin cultures as would be expected if Kitl were an essential substrate of ADAMTS20. As further evidence that the *bt* phenotype is not due to defective Kitl cleavage, *bt^9J^/bt^9J^* exhibited similar genetic interactions with both a *Kitl* null allele and with *KitL^Sl-d^/+* animals, which produce a short sKitl [42],[43],[70]. Kunisada et. al have also shown that transgenic mice over-expressing *Kitl* transgene, which greatly reduces the white spotting associated with *Kit* mutants, only slightly reduces the spotting in *bt^9J^/bt^9J^* animals [71]. Taken together this suggests that the *bt* phenotype is not due to defective cleavage of Kitl. Instead, *Adamts20* may regulate the interaction of Kitl with Kit, the activation of Kit, or signaling downstream of the receptor. Future biochemical studies will be necessary to define the exact mechanism by which *Adamts20* modulates Kit signaling.

The observation that melanoblasts in *bt^9J^/bt^9J^* trunk explants did not exhibit any response to soluble Kitl indicates that Kit signaling is defective, but it is important to note that this does not exclude the possibility that *Adamts20* may additionally regulate other factors essential for melanoblast development. For example, *Adamts20* could be required to activate other pathways that act synergistically with Kit to regulate melanoblast survival.

### ECM Alterations in *bt^9J^/bt^9J^* Mice

Another possible mechanism by which *Adamts20* could modulate survival is by altering the extracellular matrix in which melanoblasts are located. We show that *bt^9J^/bt^9J^* mutants exhibit reduced cleavage of at least one ECM component, versican. *Versican*, *Adamts20,* and *Kitl* are expressed in embryonic skin at appropriate times and sites to regulate melanoblast development [12],[24],[72]. In fact, sKitl enhances melanoblast proliferation to a greater extent when primary NC cells are cultured on chondroitin sulfate, suggesting that the ECM can modulate Kit signaling [73]. Taken in context of this study, it could be melanoblasts in *bt^9J^/bt^9J^* explant cultures did not respond to sKitl because the ECM was defective.

Versican proteolysis could influence melanoblast behavior and Kit signaling by promoting direct interactions of Kitl with Kit receptor. Such a scenario is analogous to heparin sulfate proteoglycans, which modulate growth factor signaling by sequestering ligand and promoting its receptor binding [74]. Studies of Weill-Marchesani syndrome strongly suggest *ADAMTS10* (GeneID: 81794) regulates TGF βsignaling through its interactions with the ECM component, fibrillin-1 (GeneID: 2200) [9]. Interestingly, versican binds extracellular cytokines, including the secreted growth factor, midkine (GeneID: 17242) [75]–[77]. Future studies will reveal if versican, Kit, and Kitl physically interact and if versican regulates Kit signaling directly.

Versican and its proteolytic products could also promote melanoblast survival through a pathway that is independent of Kit signaling. *Versican V1* promotes survival of NIH3T3 cells and down-regulates expression of the pro-apoptotic protein *Bad* (GeneID: 12015) [78]. Expression of the versican G1 domain protects sarcoma cells from apoptosis, and binding of the versican G3 domain to β-Integrin promotes survival of astrocytoma cells [79],[80]. Since integrins are expressed by melanoblasts [81],[82], versican-integrin interactions are another potential mechanism by which versican could influence melanoblast development. Since *versican* mutants are embryonic lethal around E10.5 [83], conditional models of *versican* as well as transgenic animals containing constitutively cleaved and uncleaved Versican molecules will be necessary to assess the requirement of *versican* for melanoblast development.

### *ADAMTS20* and Human Disease

Given that cell survival is an integral component of melanoma progression, *ADAMTS9* and *ADAMTS20* are excellent candidates for participating in melanoma. *ADAMTS20* over-expression has not been carefully examined in melanoma, but has been observed in brain, colon and breast tumors [47],[84]. The related metalloproteases *ADAM9* (GeneID: 8754) and *ADAMTS13* are upregulated in primary melanoma tumors and in melanoma cell lines, respectively [85],[86]. Interestingly, *VERSICAN* and *KITL* overexpression are observed in primary melanomas and levels correlate with melanoma progression [87]–[91]. Given the requirement of *Adamts20* for Kit signaling and versican cleavage, it is intriguing to consider how dysregulation of signaling between ADAMTS20, Kit, and versican might contribute to melanoma progression.

## Materials and Methods

### Mice and Genetic Interactions

The following mouse stocks were used and kindly provided by: *Adamts20^bt-Bei1^* (David Beier, Harvard Medical School), *Adamts20^bt9J^* (Lynn Lamoreux, Texas A&M University), *Dct-*LacZ, *Mi^Mi^, Kit^tm1Alf^*, *Kitl^Sl^* (Heinz Arnheiter, NINDS), *Kit^Sl-d^* (Jackson Laboratories, Bar Harbor, ME). *Adamts9^ko^* and Adamts5*^ko^* knockout animals (both on a C57Bl/6 background) are described elsewhere (D. McCullough, H. Enomoto, S. Apte, unpublished). Quantification of white spotting was performed using Image J software (http://rsb.info.nih.gov/ij) (NIH, Bethesda, MD) and statistical significance of spotting calculated using ANOVA tests.

### Genotyping and Sequencing

cDNA was sequenced by GeneDx (Gaithersburg, MD). *Adamts20^bt9J^* animals were genotyped using standard conditions on an ABIPrism7000 using a Taqman^TM^ assay of genomic DNA. For this assay, a region containing the point mutation is amplified by PCR. Two fluorescently labeled single-stranded oligonucleotides (probes), one complementary to the wild-type product, and one complementary to the mutant product are included in the assay. The relative amount of wild-type and mutant product is measured by fluorescence quenched upon DNA synthesis. Allelic discrimination was performed to detect the total levels of each allele at the conclusion of the PCR reaction. The following cycling conditions were used: 1. 95°C for 10 minutes 1×, 2. 92°C for 15 seconds, 3. 55°C for 60 seconds (2 and 3 repeated 40 times). The following primers and probes were used: TTCAGCACAGCTATTCTGGAAGAC (forward primer), GCACCTGAGGCAGACATACAC (reverse primer), CTTACCGAGATAGTTGTC (VIC probe, C57BL/6 allele), and CTTACCGAAATAGTTGTC (FAM probe, *bt^9J^* allele) (designed using Applied Biosystems software, Foster City, CA).

### Melanoblast Quantitation

For quantitation of whole mount embryos, melanoblasts were counted from both sides of each embryo within a 1 mm×0.7 mm field surrounding the eye or the trunk. For quantitation of melanoblasts in E13.5 sections, *Dct*-LacZ expression was scored. The mitotic index was calculated using the average number of PH3+ melanoblasts per section divided by the average number of total melanoblasts per section and multiplied by 100. The apoptotic index was calculated using the average number of CC3+ melanoblasts per section divided by the average number of total melanoblasts per section and multiplied by 100. For quantitation of melanoblasts in different skin compartments the following criteria were used: melanoblasts above the basal membrane were classified as epidermal, melanoblasts below the basal membrane were classified as dermal, and melanoblasts crossing the basal membrane and present in both regions were classified as dermal/epidermal region. The following statistical tests were used: for average number of melanoblasts in sections and whole mount embryos (two-tailed Student's t-test), for proportion of total melanoblasts and apoptotic melanoblasts in skin layers (Chi-square test of independence), for proportion of proliferating and apoptotic melanoblasts (Fisher's test).

### Whole Mount β-Galactosidase Staining and *In situ* Hybridization

β-galactosidase activity staining was performed as previously described [41]. *In situ* hybridization was performed as previously described [92] using a 1 Kb mouse *Adamts20* probe [12], a 644 bp mouse *Versican V1* probe made using mouse *Versican V1* cDNA (a kind gift of Andrew Copp, UCL), a *Pmel17* probe made as previously described [92], and a 1 Kb mouse *Adamts9* probe [58].

### Immunofluorescence

Frozen sections were prepared from embryos fixed overnight in 4% paraformaldehyde. Staining was performed with modifications to manufacturer's protocol (Vector Laboratories, Burlingame, CA), and sections were mounted using Hardset Vectashield with DAPI (Vector Laboratories). The following antibodies were used at 1∶200: mouse anti- β galactosidase (Promega, Madison, WI), rabbit anti-cleaved Caspase-3 (Cell Signaling, Danvers, MA), rabbit anti-phospho-Histone H3 (Upstate Biotechnology, Charlottesville, VA), rabbit anti-versican GAG β (Chemicon, Temecula, CA), rabbit anti-versican V0/V1 Neo (Affinity Bioreagents, Golden, CO), anti-mouse and anti-rabbit rhodamine-conjugated or FITC-conjugated secondary antibodies (Vector Laboratories).

### Generation of Expression Plasmids and Versican Digestion

*Adamts20* short isoform cDNA was assembled by PCR from E17.5 mouse embryo mRNA, sequence verified, and cloned into pcDNAmyc-hisA+ plasmid (Invitrogen, Thousand Oaks, CA). The expression plasmid for full-length *Adamts9* was previously described [47]. Transfected or untransfected cells were incubated with versican as previously described [47]. Extracts of embryo skin were prepared using a RIPA lysis buffer containing protease inhibitor mixture (20 µl) and 2 mM phenylmethylsulphonyl fluoride (Pierce, Rockford, IL). For analysis of total versican, cell extracts were treated with 0.1 units chondroitinase ABC (Associates of Cape Cod, East Falmouth, MA) in 0.1 M Tris, 50 mM NaAc for 10 minutes at 37°C. Skin extracts and conditioned medium from transfected cells were run on 4–12% SDS-Polyacrylamide gels (Invitrogen) and analyzed by Western blotting using the following primary antibodies: mouse anti-myc 9E10 (Invitrogen), rabbit anti-versican GAG βat 1∶1000, rabbit anti-versican V0/V1 Neo at 1∶1000, and mouse anti-α-Tubulin at 1∶1000 (Sigma, St.Louis, MO); and secondary antibodies: anti-rabbit HRP and anti-mouse HRP (Amersham, Piscataway, NJ).

### *Ex vivo* Embryonic Skin Analysis

Dorsal skin between the forelimb and hindlimb was isolated from E13.5 *bt/+;Dct-*LacZ and *bt/bt;Dct-*LacZ embryos with ventral tissue portions and internal organs removed. The skin explants were placed epidermal side up onto polyethylene terepthlate track-etched membranes in a cell culture insert (8.0 µm pore size, Becton-Dickinson, Franklin Lakes, NJ). Separate cultures were established for each explant. Explants were cultured for four days with 95% DMEM, 5% FBS (control medium) and as noted the medium was supplemented daily with 500 ng/ml Kitl (R&D Systems, Minneapolis, MN). After a culture period of four days, the explants were fixed in 4% paraformaldehyde in PBS (pH 7.4) for one hour and staining for β-galactosidase activity was performed as previously described [41].

### Newborn Skin Analysis

Skin was removed from newborn pups, fixed in 4% paraformaldehyde overnight and washed 3× in PBS and then analyzed on the dermal side for pigmentation. Pigmentation was scored as the extent of pigmentation between anterior and posterior edges of samples, using the following scale: 1 (no pigmentation), 2 (between 0 and 25%), 3 (between 25 and 50%), 4 (between 50 and 75%), 5 (between 75% and 100%). Statistical significance was calculated using a student's t test.

## Supporting Information

Figure S1 Melanoblast distribution is normal in bt9J/bt9J E11.5 embryos. Images of β-galactosidase stained E11.5 bt9J/+;Dct-LacZ (A,C) and bt9J/bt9J;Dct-LacZ (B,D) embryos. Shown are whole embryos (A,B) and trunks (C,D). The scale bars are: (A,B) 2 mm, and (C,D) 1 mm.
(8.94 MB TIF)

Figure S2 Melanoblasts do not build up at the lateral edges of the belt in bt9J/bt9J E16.5 embryos. (A–D) Images of the belt region of β-galactosidase stained E16.5 whole mount bt9J/+;Dct-LacZ (A,C) and bt9J/bt9J;Dct-LacZ (B,D) embryos. Shown are low magnification (A,B) and high magnification(C,D) images from four different embryos. The scale bars are (A,C) 1 mm, and (B,D) 0.5 mm.
(10.38 MB TIF)

Figure S3 Kit heterozygous mutations do not exacerbate the bt phenotype at E12.5. Representative images of the trunks of E12.5 bt9J/+;Kittm1Alf/+ (A) and bt9J/bt9J;Kittm1Alf/+ embryos (B) (n = 6 each genotype). Melanoblasts are marked using LacZ, which is targeted to the Kittm1Alf locus. The scale bar is 1 mm.
(9.47 MB TIF)
