# Supplementary figures and images for: The Secreted Metalloprotease ADAMTS20 Is Required for Melanoblast Survival
Source: PLoS Genet. 2008 Feb 29;4(2):e1000003. doi: 10.1371/journal.pgen.1000003 (PMC2265537; doi:10.1371/journal.pgen.1000003)

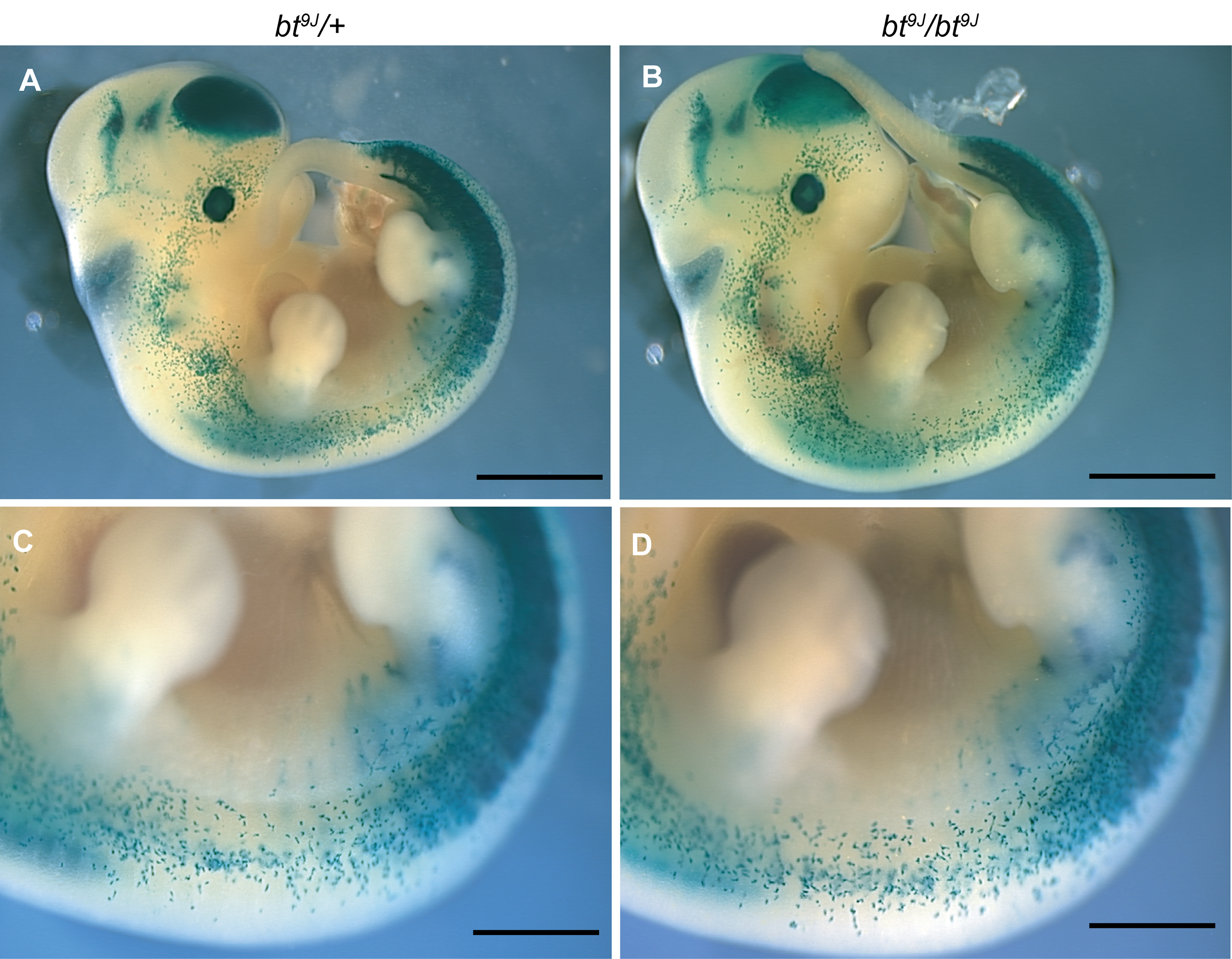

Supplement: Figure S1 — Melanoblast distribution is normal in bt9J/bt9J E11.5 embryos. Images of β-galactosidase stained E11.5 bt9J/+;Dct-LacZ (A,C) and bt9J/bt9J;Dct-LacZ (B,D) embryos. Shown are whole embryos (A,B) and trunks (C,D). The scale bars are: (A,B) 2 mm, and (C,D) 1 mm. (8.94 MB TIF) [file pgen.1000003.s001.tif]

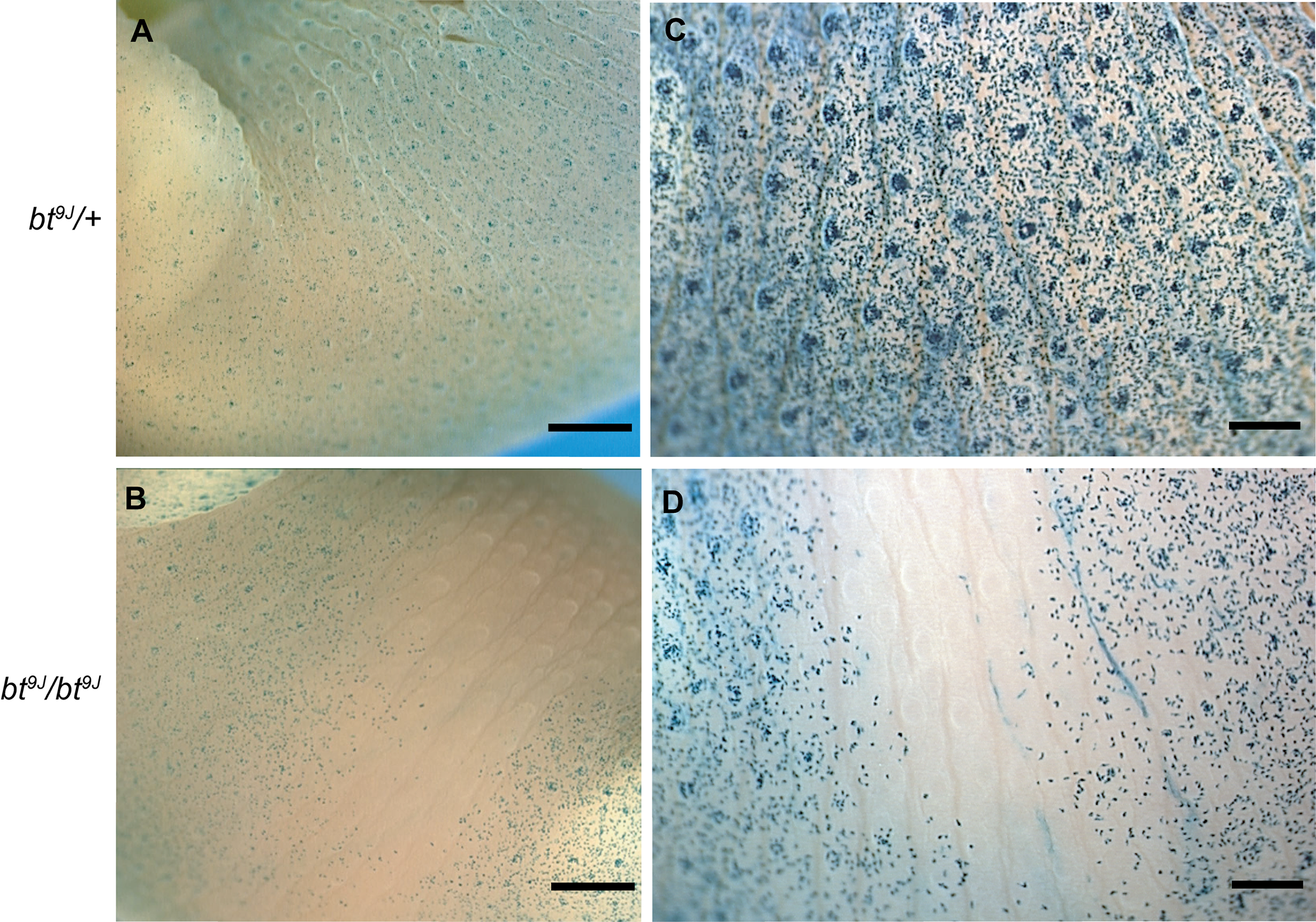

Supplement: Figure S2 — Melanoblasts do not build up at the lateral edges of the belt in bt9J/bt9J E16.5 embryos. (A–D) Images of the belt region of β-galactosidase stained E16.5 whole mount bt9J/+;Dct-LacZ (A,C) and bt9J/bt9J;Dct-LacZ (B,D) embryos. Shown are low magnification (A,B) and high magnification(C,D) images from four different embryos. The scale bars are (A,C) 1 mm, and (B,D) 0.5 mm. (10.38 MB TIF) [file pgen.1000003.s002.tif]

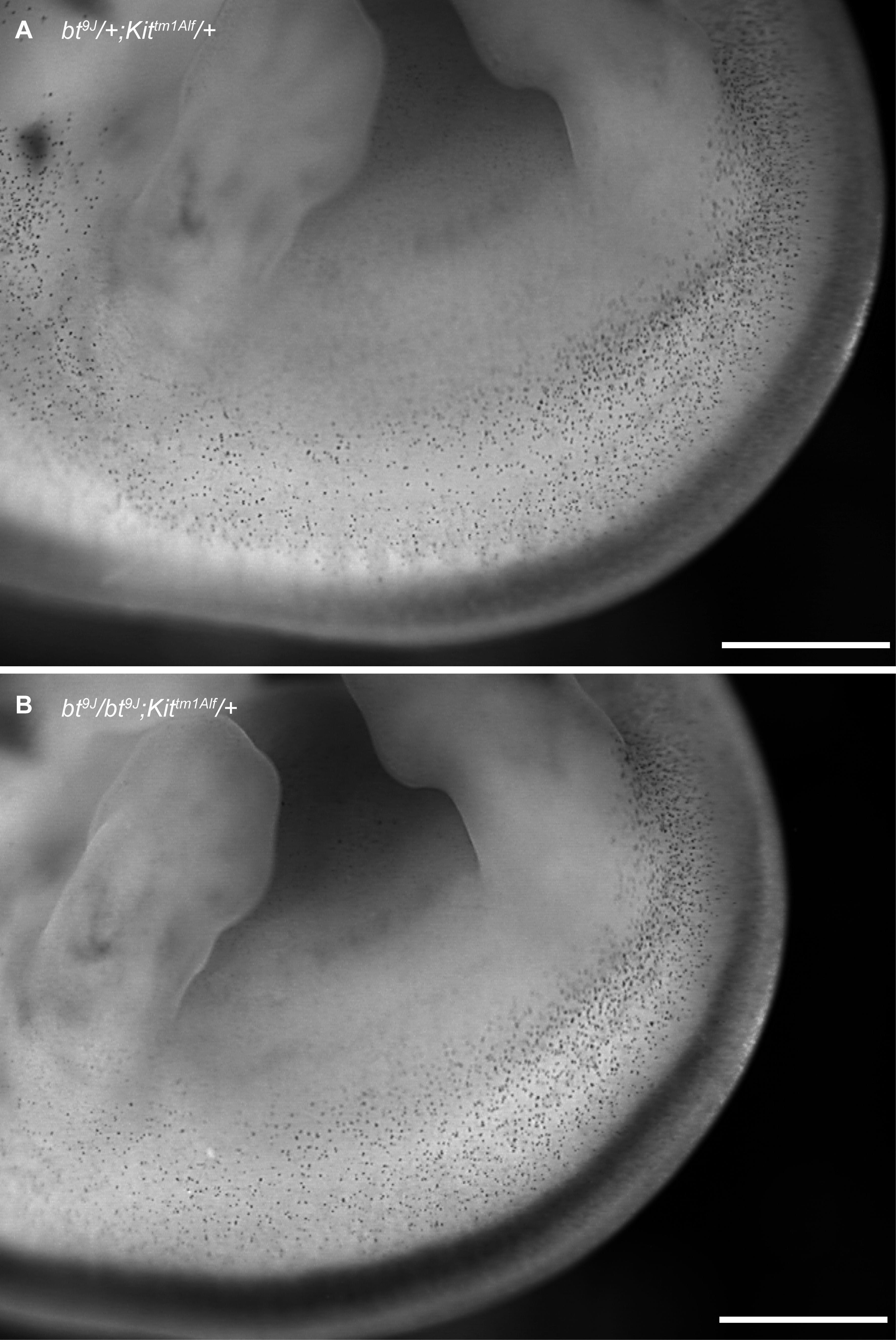

Supplement: Figure S3 — Kit heterozygous mutations do not exacerbate the bt phenotype at E12.5. Representative images of the trunks of E12.5 bt9J/+;Kittm1Alf/+ (A) and bt9J/bt9J;Kittm1Alf/+ embryos (B) (n = 6 each genotype). Melanoblasts are marked using LacZ, which is targeted to the Kittm1Alf locus. The scale bar is 1 mm. (9.47 MB TIF) [file pgen.1000003.s003.tif]
